# BRCA1 Interacts with Smad3 and Regulates Smad3-Mediated TGF-*β* Signaling during Oxidative Stress Responses

**DOI:** 10.1371/journal.pone.0007091

**Published:** 2009-09-21

**Authors:** Huchun Li, Masayuki Sekine, Seyha Seng, Shalom Avraham, Hava Karsenty Avraham

**Affiliations:** Division of Experimental Medicine, Beth Israel Deaconess Medical Center and Harvard Medical School, Boston, Massachusetts, United States of America; Health Canada, Canada

## Abstract

**Background:**

BRCA1 is a key regulatory protein participating in cell cycle checkpoint and DNA damage repair networks. BRCA1 plays important roles in protecting numerous cellular processes in response to cell damaging signals. Transforming growth factor-beta (TGF-β) is a potent regulator of growth, apoptosis and invasiveness of tumor cells. TFG-β activates Smad signaling via its two cell surface receptors, the TbetaRII and ALK5/TbetaRI, leading to Smad-mediated transcriptional regulation.

**Methodology/Principal Findings:**

Here, we report an important role of BRCA1 in modulating TGF-β signaling during oxidative stress responses. Wild-type (WT) BRCA1, but not mutated BRCA1 failed to activate TGF-β mediated transactivation of the TGF-β responsive reporter, p3TP-Lux. Further, WT-BRCA1, but not mutated BRCA1 increased the expression of Smad3 protein in a dose-dependent manner, while silencing of WT-BRCA1 by siRNA decreased Smad3 and Smad4 interaction induced by TGF-β in MCF-7 breast cancer cells. BRCA1 interacted with Smad3 upon TGF-β1 stimulation in MCF-7 cells and this interaction was mediated via the domain of 298–436aa of BRCA1 and Smad3 domain of 207–426aa. In addition, H_2_O_2_ increased the colocalization and the interaction of Smad3 with WT-BRCA1. Interestingly, TGF-β1 induced Smad3 and Smad4 interaction was increased in the presence of H_2_O_2_ in cells expressing WT-BRCA1, while the TGF-β1 induced interaction between Smad3 and Smad4 was decreased upon H_2_O_2_ treatment in a dose-dependent manner in HCC1937 breast cancer cells, deficient for endogenous BRCA1. This interaction between Smad3 and Smad4 was increased in reconstituted HCC1937 cells expressing WT-BRCA1 (HCC1937/BRCA1). Further, loss of BRCA1 resulted in H_2_O_2_ induced nuclear export of phosphor-Smad3 protein to the cytoplasm, resulting decreased of Smad3 and Smad4 interaction induced by TGF-β and in significant decrease in Smad3 and Smad4 transcriptional activities.

**Conclusions/Significance:**

These results strongly suggest that loss or reduction of BRCA1 alters TGF-β growth inhibiting activity via Smad3 during oxidative stress responses.

## Introduction

Patients who inherit genetic defects in BRCA1 and BRCA2 have an increased lifetime risk of developing breast cancer. BRCA1 is a multifunctional protein that has been implicated in many cellular processes, including genomic stability, the cell-cycle checkpoint, DNA-damage repair, apoptosis, and gene transcription [1]. However, the precise mechanism by which loss of BRCA1 affects specific tissues in humans is unclear. BRCA1 has two important structural motifs, including a highly conserved amino-terminal RING finger motif and tandem BRCT motifs at its C-terminus [2], [3]. The RING finger motif confers BRCA1 E3 ubiquitin ligase activity, one of the intriguing aspects of BRCA1 function, regulating activity, stability and distribution of target molecules [4]. The BRCT region of BRCA1 is essential for its DNA repair, transcriptional regulation and tumor suppressor functions [5]. Germline mutations in BRCA1 were often seen in the two regions [6], suggesting that the RING finger and BRCT motifs play an important role in the development of breast and ovarian cancers.

Emerging evidence has indicated that BRCA1 is involved in ROS production and oxidative stress responses. BRCA1 was shown to exert antioxidant activity by inducing antioxidant expression [7] and Brca1-deficient mice were reported to produce excess reactive oxygen species (ROS) and to be sensitive to oxidative stress [8]. However, the molecules and signaling pathways susceptible to oxidative stress due to BRCA1 inactivation remain elusive. Studies have suggested that oxidative stress and ROS play important roles in the development of cancer [9], [10]. ROS can serve as subcellular messengers in gene regulation and signal transduction pathways, and can damage lipids, DNA and proteins [11], [12]. Therefore, understanding the targets and signaling pathways of ROS may provide insights into the impact of environmental factors in cancer initiation and progression.

The TGF-*β* family has been demonstrated to contribute to normal mammary gland development as well as the progression of human breast cancer [13]. Loss of inhibition or increased promotion of proliferation for TGF-*β* is believed to contribute to carcinogenesis in the mammary gland. TGF-*β* transduces signals via phosphorylation of intracellular mediators, Smad2 and Smad3. The TGF-*β* receptor-activated Smads form a complex with Smad4 co-activator. The heteromeric Smad complexes then translocate into the nucleus, where they induce or repress transcription of defined genes [14]. Smad-dependent downregulation of c-Myc or upregulation of p15 and p21 is related to the anti-proliferative activity of TGF-*β* [15]. TGF-*β* also signals independently of Smads, via phosphatidylinositol 3-kinase (PI3K), protein phosphatase 2A/p70 S6 kinase (PP2A/p70S6K), and various mitogen-activated protein kinase (MAPK) pathways, which may result in promotion of proliferation [16]. Whether and how genetic defects and/or environmental factors influence the growth inhibitory activity of TGF-*β* signaling is not well understood.

TGF-*β* signaling, via Smad3, was reported to suppress BRCA1-dependent DNA repair in response to DNA-damaging agents [17]. Here, we aimed to elucidate the effects of BRCA1 inactivation on TGF-*β* signaling. Since ROS can affect cell fate through cross-talk with other signaling pathways [18], we investigated the functional interaction between BRCA1 and Smad3 during cross-talk between TGF-*β* signaling and oxidative stress responses. We describe that the oxidative stress reagent H_2_O_2_ increases the TGF-*β*-induced association between BRCA1 and Smad3 *in vivo* and that inactivation of BRCA1 of loss of BRCA1 sensitizes phospho-Smad3 protein to oxidative stress resulting in nuclear export of phospho-Smad3 protein, and decreased Smad3-Smad4 mediated interaction induced by TGF-β and transcriptional activation in breast cancer cells.

## Results

### BRCA1 activates a TGF-β-responsive reporter

**Figure 1:**
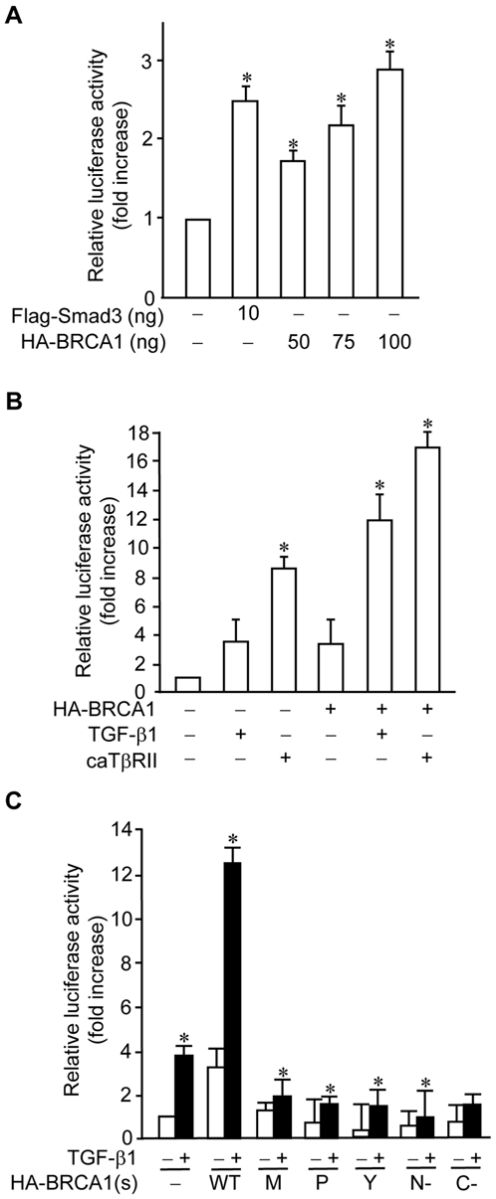
Effects of BRCA1 on p3TP-Lux promoter activity. (A) Wild type BRCA1 activates the p3TP-Lux promoter. 0.1 µg of p3TP-Lux and 10 ng of pCMV*β*-galactosidase plasmids were co-transfected with Flag-Smad3 and HA-BRCA1 plasmids into COS-7 cells, as indicated. A pcDNA3 empty vector plasmid was used to adjust total DNA amounts. At 36 hrs after transfection, luciferase and *β*-galactosidase assays were performed. The luciferase activities were normalized to *β*-galactosidase activity and the relative luciferase activities (fold increase) were calculated as the ratio of the normalized luciferase activity with effectors to that without effectors. The average±S.D. was calculated from triplicate plates and experiments were repeated three times with similar results. The bars in the graphs represent mean±SD. *, *P*<0.05 versus empty vector only. (B) Wild type BRCA1 enhances TGF-*β*-mediated p3TP-Lux promoter activity. 0.1 µg of p3TP-Lux and 10 ng of pCMV*β*-galactosidase plasmids were co-transfected with 0.25 µg of caT*β*RII plasmid or 0.1 µg of HA-BRCA1 plasmid into COS-7 cells, as indicated. 24 hrs after transfection, the indicated cells were incubated with 2 ng/ml of TGF-*β*1 ligand overnight, followed by luciferase and *β*-galactosidase assays. The luciferase activities were normalized to *β*-galactosidase activity and the relative luciferase activities (fold increase) were calculated as the ratio of the normalized luciferase activity with effectors to that without effectors. The average±S.D. was calculated from triplicate plates and experiments were repeated three times with similar results. The bars in the graphs represent mean±SD. (C) BRCA1 mutants could not activate the p3TP-Lux promoter. 0.1 µg of p3TP-Lux and 10 ng of pCMV*β*-galactosidase were co-transfected with 0.1 µg of wild type or various mutant forms of HA-BRCA1 into COS-7 cells. 24 hrs after transfection, the cells were treated with or without 2 ng/ml of TGF-β1 overnight, followed by luciferase and *β*-galactosidase assays. The luciferase activities were normalized to *β*-galactosidase activity and the relative luciferase activities (fold increase) were calculated as the ratio of the normalized luciferase activity with effectors to that without effectors. The average±S.D. was calculated from triplicate plates and experiments were repeated three times with similar results. WT: wild type BRCA1; M, P, and Y: BRCA1-M1775R, BRCA1-P1749R, and BRCA1-Y1853x, respectively; N-: BRCA1 (1–683aa); C-: BRCA1 (1301–1863aa).

To understand whether inactivation of BRCA1 by germline mutations affects the TGF-*β* signaling pathway, we investigated the effect of BRCA1 mutations on TGF-*β*1 transactivation activity by performing a cell-based reporter assay with a TGF-*β*-responsive reporter, p3TP-Lux [19]. We found that wild type BRCA1 increases the basal level of p3TP-Lux reporter activity in a dose-dependent manner in COS-7 cells (Figure 1A). We further examined the effect of wild type BRCA1 on TGF-*β*1-mediated transcriptional regulation. TGF-*β*1-mediated transactivation of the p3TP-Lux reporter was increased by wild type BRCA1 (Figure 1B). Similarly, wild type BRCA1 augmented constitutively active form of TGF-*β* receptor II (caT*β*RII)-mediated transactivation activity (Figure 1B). To understand how BRCA1 mutations affect TGF-*β*1-mediated transactivation, we examined several mutants of BRCA1 that are frequently seen in BRCA1 familial breast cancer patients [20]. Intriguingly, mutants of BRCA1, including BRCA1-M1775R, BRCA1-P1749R, BRCA1-Y1853x, BRCA1 (1–683aa), and BRCA1 (1301–1863aa), not only failed to induce the basal activity of the p3TP-Lux reporter, but also failed to increase the TGF-*β*1-mediated activation of the reporter (Figure 1C). Thus, these data suggest that wild type BRCA1 upregulates TGF-*β*1-mediated transcription, which is impaired upon inactivation of BRCA1 by germline mutations.

**Figure 2:**
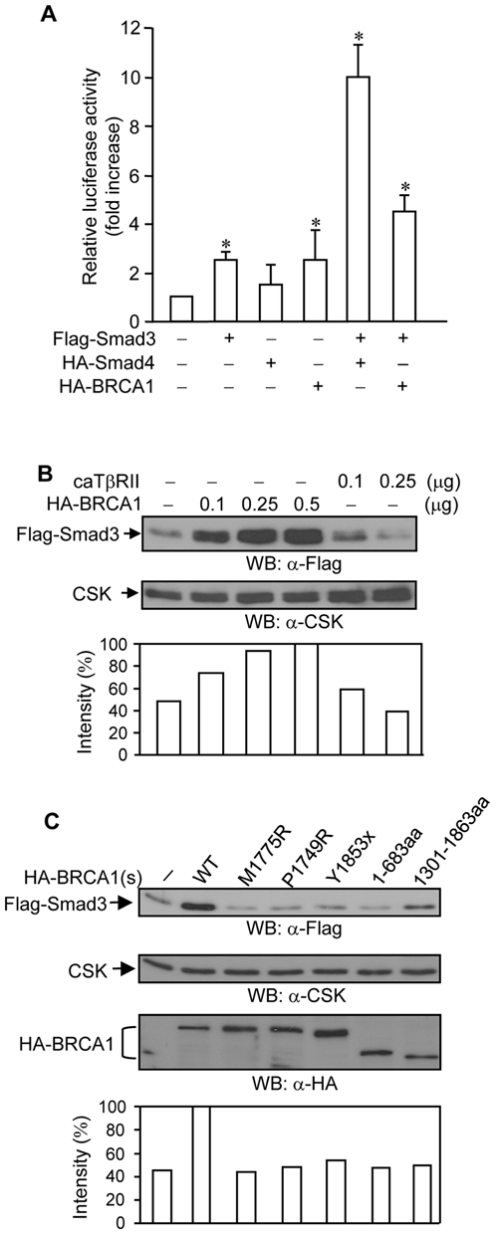
Effects of BRCA1 on Smad3 protein. (A) Wild type BRCA1 and Smad3 cooperate to increase p3TP-Lux promoter activity. 0.1 µg of p3TP-Lux and 10 ng of pCMV*β*-galactosidase plasmids were co-transfected with 10 ng of Flag-Smad3, 50 ng of HA-Smad4, and 0.1 µg of wild type HA-BRCA1 plasmids into COS-7 cells, as indicated. 36 hrs after transfection, luciferase and *β*-galactosidase assays were performed. The luciferase activities were normalized to *β*-galactosidase activity and the relative luciferase activities (fold increase) were calculated as the ratio of the normalized luciferase activity with effectors to that without effectors. The average±S.D. was calculated from triplicate plates and experiments were repeated three times with similar results. The bars in the graphs represent mean±SD. *, *P*<0.05 versus empty vector only. (B) Wild type BRCA1 increases Smad3 protein abundance. 0.15 µg of Flag-Smad3 plasmid was co-transfected with 0.1 µg, 0.25 µg, and 0.5 µg of wild type HA-BRCA1 or 0.1 µg and 0.25 µg of caT*β*RII into HEK293 cells. 36 hrs after transfection, 10 µg of total cell lysates was subjected to Western blotting with anti-Flag monoclonal antibody. Anti-CSK polyclonal antibody was used to monitor equal loading. Flag-Smad3 expression levels were normalized to CSK and are represented graphically. This is a representative experiment out of 4 experiments. (C) BRCA1 mutants could not increase Smad3 protein abundance. 0.15 µg of Flag-Smad3 was co-transfected with 0.1 µg of wild type or various mutant forms of HA-BRCA1 into HEK293 cells. 10 µg of total cell lysates was subjected to Western blotting with anti-Flag monoclonal antibody. Anti-CSK polyclonal antibody was used to monitor equal loading. Flag-Smad3 expression levels were normalized to CSK and are represented graphically. This is a representative experiment out of 4 experiments.

It has been demonstrated that BRCA1 increases Smad3-dependent transcription [17]. We also observed that wild type BRCA1 and Smad3 cooperate to increase the luciferase activity of the p3TP-Lux reporter (Figure 2A), while Smad4 largely increases the Smad3-dependent transactivation activity of the reporter (Figure 2A).

Lysates from the p3TP-Lux reporter assay were examined for the expression of Flag-Smad3 protein. Interestingly, the expression levels of Flag-Smad3 protein were increased in conjunction with wild type BRCA1 co-expression (data not shown). Wild type BRCA1 increased the expression levels of Flag-Smad3 protein in a dose-dependent manner, as compared to the control caT*β*RII (Figure 2B). Notably, the BRCA1 mutants could not increase the expression levels of Smad3 protein (Figure 2C). Taken together, these data suggest that an increase in the expression levels of Smad3 protein by wild type BRCA1 may contribute to increased Smad3-mediated reporter activation.

### Interaction of endogenous WT-BRCA1 with Smad3

**Figure 3:**
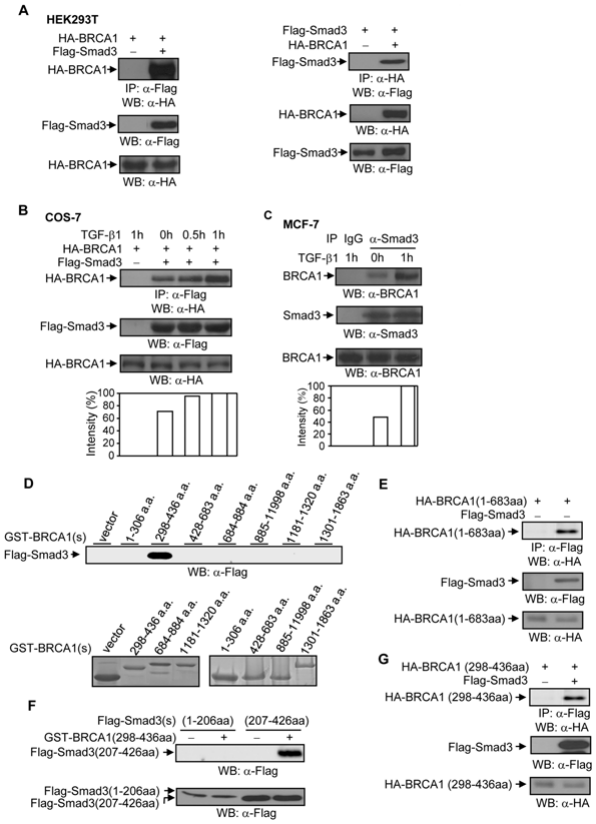
BRCA1 and Smad3 association. (A) HA-BRCA1 interacts with Flag-Smad3 in HEK293T cells. 0.5 µg of wild type HA-BRCA1 and 0.50 µg of Flag-Smad3 plasmids were co-transfected into HEK293T cells, as indicated. 36 hrs later, total cell lysates were subjected to immunoprecipitation and Western blotting with anti-Flag monoclonal antibody and anti-HA-monoclonal antibody, as indicated. 10 µg of total cell lysates was used to examine the expression levels of Flag-Smad3 and HA-BRCA1 protein. The blots are representative experiment out of 3 experiments. (B) TGF-*β*1 increases the interaction between HA-BRCA1 and Flag-Smad3 in COS-7 cells. 0.50 µg of Flag-Smad3 and 0.50 µg of HA-BRCA1 plasmids were co-transfected into COS-7 cells. 24 hrs later, cells were treated with or without 2 ng/ml of TGF-*β*1 as indicated. Total cell lysates were immunoprecipitated with anti-Flag monoclonal antibody followed by Western blotting with anti-HA monoclonal antibody. The membrane was re-probed with anti-Flag antibody. The increases in HA-BRCA1 were normalized to Flag-Smad3 and are represented graphically. The expression levels of HA-BRCA1 protein were detected in 10 µg of total cell lysates. This is a representative experiment out of 3 experiments. (C) TGF-*β*1 increases BRCA1 and Smad3 interaction in MCF-7 cells. Cells were stimulated with 2 ng/ml of TGF-*β*1 for 1 hour. Total cell lysates were immunoprecipitated by anti-Smad3 polyclonal antibody, followed by Western blotting with anti-BRCA1 antibody. The membrane was re-probed with anti-Smad3 antibody. The increases in BRCA1 were normalized to Smad3 and are represented graphically. The expression levels of BRCA1 protein were determined in 30 µg of total cell lysates. This is a representative experiment out of 4 experiments. (D) Smad3 binding site in BRCA1. 10 µg of the individual GST-BRCA1 protein fragments and HEK293T cell lysates transfected with Flag-Smad3 plasmid were subjected to a GST-pull down assay. To monitor expression levels, 10 µg of individual GST-BRCA1 protein fragments were separated on 15% and 8% SDS-PAGE, followed by Coomassie staining. (E) Interaction of BRCA1 (1–683aa) with Smad3 protein in HEK293T cells. 0.5 µg of HA-*BRCA1*(1–683aa) and 0.5 µg of *Smad3* plasmids were co-transfected into HEK293T cells, followed by immunoprecipitation and Western blotting, as indicated. The membrane was re-probed with anti-Flag monoclonal antibody to examine the levels of Smad3 protein expression. The levels of HA-BRCA1(1–683aa) expression were determined in 10 µg of total lysates. (F) BRCA1 binds to the MH2 domain in Smad3. 10 µg of GST and GST-BRCA1 (298–436aa) proteins and HEK293T cell lysates transfected with Flag-Smad3 (1–206aa) or Flag-Smad3 (207–426aa) were subjected to a GST pull-down assay. The expression levels of Flag-Smad3 (1–206aa) and Flag-Smad3 (207–426aa) protein were determined in 10 µg of total cell lysates. This is a representative experiment out of 4 experiments. (G) Flag-Smad3 and HA-BRCA1 (298–436aa) interaction. HA-BRCA1 (298–436aa) and Flag-Smad3 plasmids were co-transfected into HEK293T cells, followed by immunoprecipitation and Western blotting, as indicated. The membrane was re-probed with anti-Flag antibody to monitor Flag-Smad3 expression. 10 µg of total cell lysates was used to examine the expression levels of HA-BRCA1 (298–436aa) protein.

An interaction between HA-BRCA1 and Flag-Smad3 was observed in HEK293T cells cotranfected with HA-BRCA1 and Flag-Smad3 (Figure 3A), consistent with a previous report [17]. TGF-*β*1 stimulation in COS-7 cells increased the association between HA-BRCA1 and Flag-Smad3 in a time-dependent manner (Figure 3B). Further, endogenous interaction of BRCA1 with Smad3 was increased with upon TGF-*β*1 stimulation in MCF-7 cells (Figure 3C). Thus, these data suggest that TGF-*β*1 regulates the association between BRCA1 and Smad3.

Next, we mapped the Smad3-binding site on the BRCA1 protein by using HEK293T cell lysates that were transfected with Flag-Smad3 plasmid and a series of GST-BRCA1 protein fragments [20]. Flag-Smad3 was shown to bind to the BRCA1 (298–436aa) protein fragment (Figure 3D). The interaction between HA-BRCA1 (298–436aa) and Flag-Smad3 was further demonstrated in HEK293T cells (Figure 3E–3G), indicating that Smad3 protein binds to one of two major protein-protein interacting surfaces in BRCA1.

Smads share two conserved regions denoted the Mad homology 1 (MH1) and MH2 domains [21], [22]. The MH1 domain of certain Smads directly binds to DNA, whereas the MH2 domain possesses intrinsic transactivation activity. To identify the BRCA1 binding site in Smad3, Flag-Smad3NL that covers MH1 and linker domains (from 1 to 206aa) and Flag-Smad3C that covers MH2 domain (from 207 to 426aa) were generated and used for the GST pull-down assay with GST-BRCA1 (298–436) protein. Flag-Smad3C was shown to bind to the GST-BRCA1 (298–436) protein (Figure 3F, 3G), suggesting that the MH2 domain, the transcriptional activation domain in Smad3 protein, mediates the association of Smad3 with BRCA1.

### Hydrogen peroxide increases the interaction between Smad3 and BRCA1 in the presence of TGF-β1

**Figure 4:**
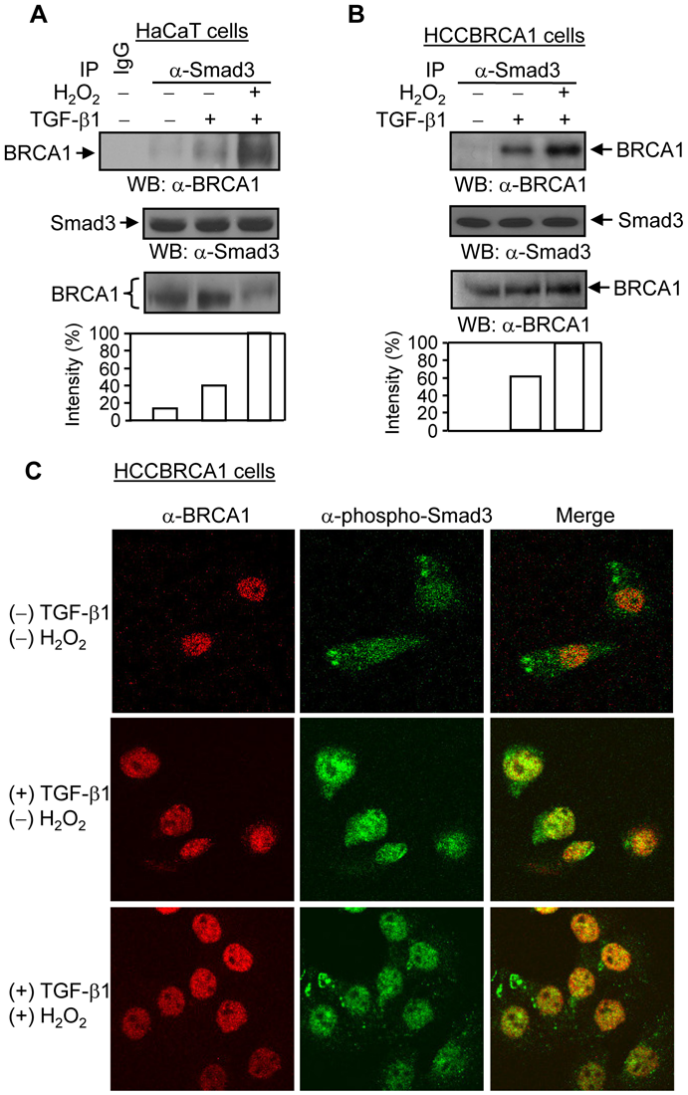
Effects of H_2_O_2_ on the association and colocalization between Smad3 and BRCA1. (A & B) H_2_O_2_ increases Smad3 and BRCA1 interaction in HaCaT and HCCBRCA1 cells. Cells were treated with TGF-*β*1 (2 ng/ml) and H_2_O_2_ (100 µM) for 1 hour, as indicated. Total cell lysates were subjected to immunoprecipitation with anti-Smad3 polyclonal antibody and Western blotting with anti-BRCA1 polyclonal antibody. The increases in Smad3-binding to BRCA1 were normalized to Smad3 protein expression and are represented graphically. The expression levels of Smad3 and BRCA1 proteins were determined in 20 and 30 µg of total cell lysates, respectively. The shifted bands of BRCA1 after H_2_O_2_ treatment are shown in HaCaT cells. This is a representative experiment out of 4 experiments. (C) H_2_O_2_ increases phospho-Smad3 and BRCA1 colocalization in HCCBRCA1 cells. HCCBRCA1 cells were treated with 2 ng/ml of TGF-*β*1 and 100 µM of H_2_O_2_ for 1 hour as indicated. Anti-BRCA1 monoclonal antibody (Ab-3) and anti-phospho-Smad3 polyclonal antibody were used to immunostain BRCA1 protein (Red) and phospho-Smad3 protein (Green), respectively. Merge shows the colocalization of BRCA1 and phospho-Smad3 protein. This is a representative experiment out of 4 experiments.

Given that BRCA1 is implicated in DNA damage-related cellular processes, we explored whether oxidative stress regulates the BRCA1 and Smad3 interaction. In the presence of TGF-*β*1, H_2_O_2_ increased the BRCA1 and Smad3 interaction at the endogenous level in human keratinocyte HaCaT cells (Figure 4A) and wild type BRCA1-reconstituted HCC1937 breast cancer cells (HCCBRCA1) (Figure 4B). The confocal analyses showed that H_2_O_2_ induces an increasing colocalization between TGF-*β*1-activated phospho-Smad3 and BRCA1 protein in HCCBRCA1 (Figure 4C) and MCF-7 cells (data not shown). In the absence of TGF-*β*1 ligand, the Smad3 and BRCA1 interaction was not induced by H_2_O_2_ in these cells (data not shown). Thus, these data suggest that oxidative stress increases the TGF-*β*1-dependent interaction between BRCA1 and Smad3.

### The TGF-β1-induced Smad3 and Smad4 interaction is regulated by H_2_O_2_ and BRCA1

**Figure 5:**
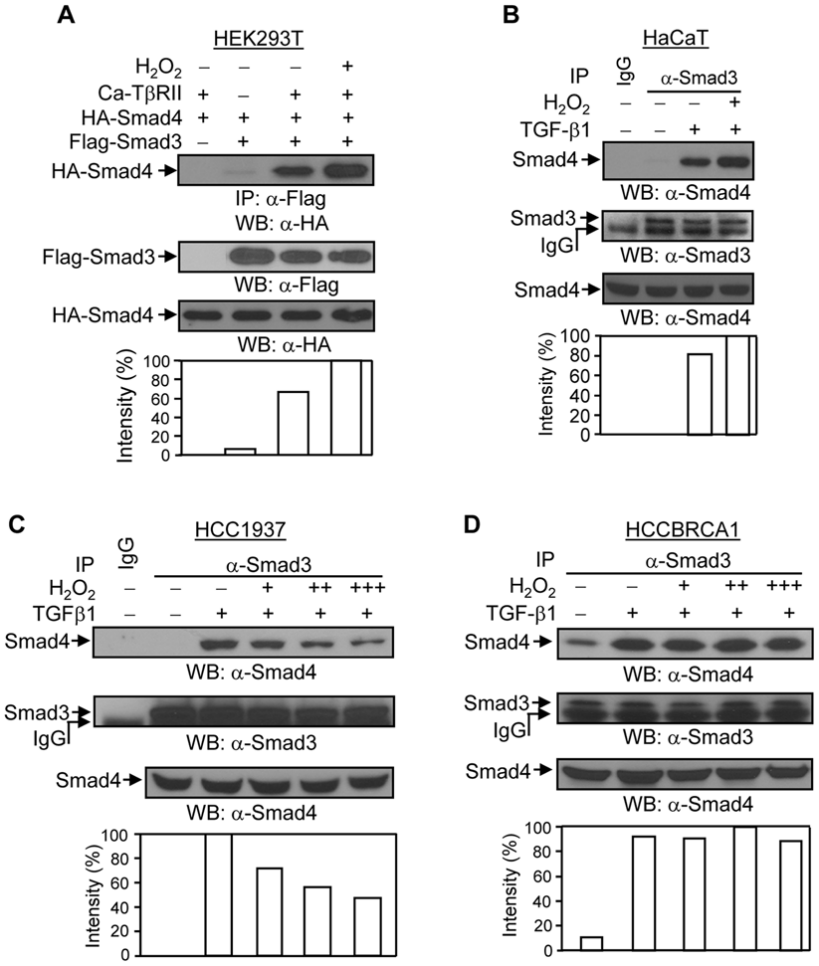
Effects of H_2_O_2_ on the TGF-*β*1-induced association between Smad3 and Smad4. (A) H_2_O_2_ increases the interaction between Smad3 and Smad4 in HEK293T cells. HEK293T cells were co-transfected with 0.5 µg of Flag-Smad3, 0.5 µg of HA-Smad4, and 0.15 µg of caT*β*RII plasmids, as indicated. 24 hours later, cells were treated with 200 µM of H_2_O_2_ for 2 hours, followed by immunoprecipitation and Western blotting, as indicated. The membrane was re-probed with anti-Flag monoclonal antibody to examine the expression levels of Flag-Smad3 protein. The increases in Flag-Smad3-binding to HA-Smad4 were normalized to Flag-Smad3 protein expression and are represented graphically. The expression levels of HA-Smad4 protein were determined in 10 µg of total cell lysates. This is a representative experiment out of 4 experiments. (B) H_2_O_2_ increases the interaction between Smad3 and Smad4 in HaCaT cells. HaCaT cells were treated or untreated with 2 ng/ml of TGF-*β*1 and 100 µM of H_2_O_2_ for 1 hour as indicated, and subjected to immunoprecipitation with anti-Smad3 polyclonal antibody and Western blotting with anti-Smad4 monoclonal antibody. The membrane was re-probed with anti-Smad3 antibody to examine the levels of Smad3 protein. The increases in Smad3-binding to Smad4 were normalized to Smad3 protein expression and are represented graphically. The expression levels of Smad4 protein were determined in 20 µg of total cell lysates. This is a representative experiment out of 4 experiments. (C) TGF-*β*1-induced Smad3 and Smad4 interaction is decreased by H_2_O_2_ in HCC1937 cells. HCC1937 cells were treated or untreated with 2 ng/ml of TGF-*β*1 and 100 (+), 200 (++), and 300 µM (+++) of H_2_O_2_ for 1 hour as indicated. Immunoprecipitation was done as in Figure 5b (HaCaT cells). The membrane was re-probed with anti-Smad3 antibody to examine the levels of Smad3 protein. The decreases in Smad3-binding to Smad4 were normalized to Smad3 protein expression and are represented graphically. The expression levels of Smad4 protein were determined in 20 µg of total cell lysates. This is a representative experiment out of 4 experiments. (D) Wild type BRCA1 restores the TGF-*β*1-induced Smad3 and Smad4 interaction against H_2_O_2_ in HCCBRCA1 cells. The experiment was done as in Figure 5c (HCC1937 cells). The increases in Smad3-binding to Smad4 were normalized to Smad3 protein expression and are represented graphically. This is a representative experiment out of 4 experiments.

We further investigated whether H_2_O_2_ affects TGF-*β*1-induced Smad3 and Smad4 interaction. In HEK293T cells, H_2_O_2_ treatment increased the caT*β*RII-induced interaction between Flag-Smad3 and HA-Smad4 (Figure 5A). In addition, H_2_O_2_ slightly increased the TGF-*β*1-dependent interaction between Smad3 and Smad4 at the endogenous level in HaCaT cells (Figure 5B). Notably, in HCC1937 cells, the TGF-*β*1-induced interaction between Smad3 and Smad4 was decreased upon H_2_O_2_ treatment in a dose-dependent manner (Figure 5C). However, HCCBRCA1 cells in the TGF-*β*1-induced increased Smad3 and Smad4 interaction (Figure 5D). The expression levels of phospho-Smad3 protein were not changed upon H_2_O_2_ treatment in either cell line (data not shown). These results suggest that loss of BRCA1 sensitizes TGF-*β*1 signaling to H_2_O_2_, leading to a decrease in the TGF-*β*1-induced Smad3 and Smad4 interaction.

### Loss of BRCA1 results in H_2_O_2_-induced nuclear export of phospho-Smad3 protein

**Figure 6:**
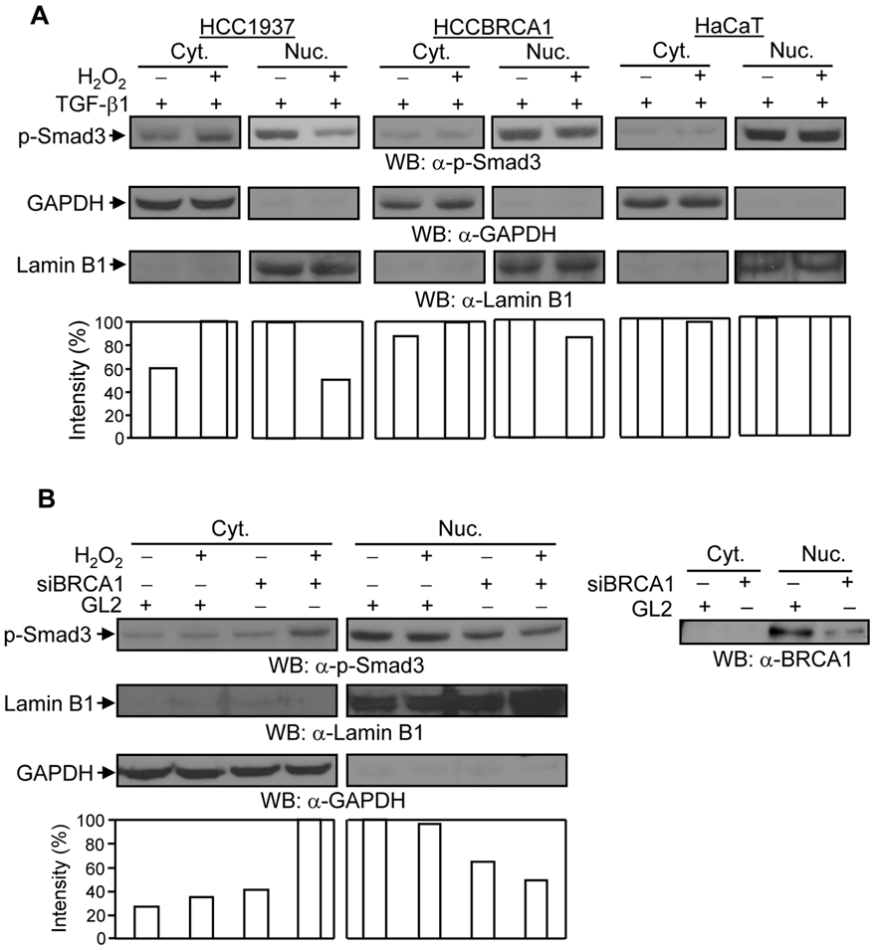
Effects of H_2_O_2_ on the trafficking of phospho-Smad3 protein. (A) H_2_O_2_ induces the nuclear export of TGF-*β*1-activated phospho-Smad3 protein (p-Smad3) in HCC1937 cells, but not in HCCBRCA1 or HaCaT cells. Cells were pretreated with 2 ng/ml of TGF-*β*1 for 1 hour and were then incubated with or without 100 µM of H_2_O_2_ for an additional 1 hour. Cytosolic and nuclear proteins were subjected to Western blotting with anti-phospho-Smad3 antibody. The membranes were re-probed with anti-GAPDH and anti-Lamin B1 antibodies to monitor fractionation efficiency and equal loading for the cytosolic and nuclear proteins. Increases or decreases in the amount of phospho-Smad3 protein were normalized to the amount of GAPDH and Lamin B1 protein in the cytosol and nucleus, respectively. Cyt.: cytosolic fraction; Nuc.: nuclear fraction. This is a representative experiment out of 4 experiments. (B) Knockdown of endogenous BRCA1 in HCCBRCA1 cells sensitizes phospho-Smad3 protein to H_2_O_2_ and induces redistribution of the protein into the cytoplasm. HCCBRCA1 cells were transfected with siRNAs against BRCA1 and GL2 (control). 48 hours later, cells were pretreated with 2 ng/ml of TGF-*β*1 for 1 hour and were then incubated with or without 100 µM of H_2_O_2_ for an additional 1 hour. Cytosolic and nuclear proteins were subjected to Western blotting with anti-phospho-Smad3 antibody (p-Smad3). The membranes were re-probed with anti-Lamin B1 and anti-GAPDH antibodies, as indicated, to monitor fractionation efficiency and equal loading. The changes in the amount of phospho-Smad3 protein were normalized to the amount of GAPDH and Lamin B1 protein in the cytosol and nucleus, respectively. The right panel shows the knockdown of nuclear BRCA1 protein after siRNA against BRCA1. Cyt.: cytosolic fraction; Nuc.: nuclear fraction. This is a representative experiment out of 4 experiments.

We further investigated whether H_2_O_2_ regulates the nuclear retention of phospho-Smad3 protein. Cells were pretreated with TGF-*β*1 for 1 hour and then incubated with or without H_2_O_2_ for an additional 1 hour. Cytosolic and nuclear proteins were separated to determine the expression levels of phospho-Smad3 protein. Phospho-Smad3 protein was translocated into the nucleus by TGF-*β*1 ligand stimulation, but not by H_2_O_2_ treatment (data not shown). Intriguingly, the TGF-*β*1-induced phospho-Smad3 protein present in the nucleus was markedly redistributed into the cytoplasm upon H_2_O_2_ treatment in HCC1937 cells, but not significantly redispersed in HCCBRCA1 and HaCaT cells (Figure 6A), or COS-7 and MCF-7 cells (data not shown). siRNA-mediated knockdown of endogenous BRCA1 in HCCBRCA1 cells resulted in a decrease in the levels of phospho-Smad3 protein in the nucleus and in a redistribution of phospho-Smad3 protein into the cytoplasm after H_2_O_2_ treatment (Figure 6B). Thus, these data suggest that loss of BRCA1 sensitizes phospho-Smad3 protein in the nucleus to H_2_O_2_-induced nuclear export.

### Effect of silenced BRCA1 on Smad3 and Smad4 interaction and transcriptional activation in breast cancer cells

**Figure 7:**
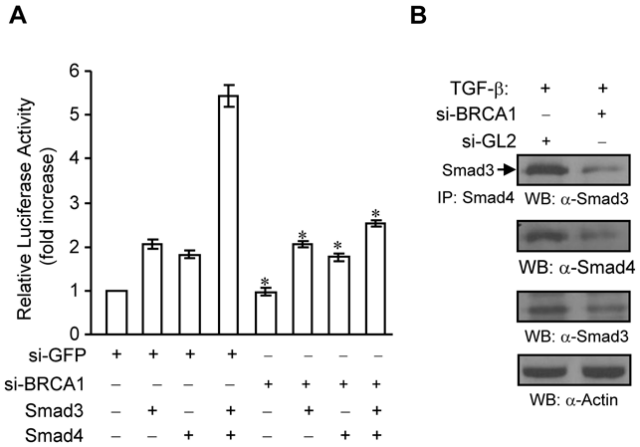
Effects of silencing BRCA1 in MCF-7 cells on Smad3 and Smad4. (A) si-BRCA1 decreases Smad3-Smad4-mediated transcriptional activation of p3TP-Lux reporter in MCF7 cells. MCF7 cells were transfected with 5 nM of siRNAs in 24 well plate. After 16–24 hours, the cells were transfected with p3TP-Lux, pCMV-β-galactosidase, Smad3, Smad4, and empty vector plasmids as indicated. The cells were maintained in cell culture medium containing 0.1% FBS overnight, followed by luciferase and β-galactosidase assays. The fold increase of luciferase activity was obtained from triplicate of three independent experiments, after being normalized by β-glactosidase activity. The Excel software was used to calculate the standard deviation. * *P*<0.05 as compared to si-GFP treatments+Smad3+Smad4. (B) Depletion of BRCA1 decreases TGF-β-induced Smad3-Smad4 interaction in MCF-7 cells. MCF-7 cells were transfected with 5 nM siGL2 or siBRCA1. After overnight, cells were starved for 3 hours, followed by 1 ng/ml of TGF-β1 treatment for 45 min. Total lysates were subjected to immunoprecipitation with anti-Smad4 antibody and Western blotting with anti-Smad3 antibody as indicated. The membrane was re-probed with anti-Smad4 antibody to monitor the levels of Smad4 protein. The levels of Smad3 protein was detected from 20 µg of total lysatesby using anti-Smad3 antibody. This membrane was reprobed with anti-Actin antibody to monitor equal loading.

Next, to examine the effects of silencing WT-BRCA1, by siRNA for BRCA1, on TGF-β mediated effects, MCF-7 breast cancer cells were either treated with si-GFP or siBRCA1. We confirmed the expression of BRCA1 to be significantly reduced by siBRCA1 in MCF7 cells (data not shown). As shown in Fig. 7a, silencing of BRCA1 significantly inhibited Smad3 and Smad4 transcriptional activation. Further, MCF-7 treated with siBRCA1 showed significant reduction of Smad3 with Smad4 TGF-β-induced interaction as well as reduced expression of Smad3 and Smad4. Taken together, these results show that loss of BRCA1 or reduced BRCA1 expression significantly modulated TGF-β induced activation of Smad3 and Smad4 in breast cancer cells.

## Discussion

In this study, we uncovered an important role of BRCA1 in the TGF-*β* signaling pathway during oxidative stress responses. BRCA1 protein associated with Smad3 protein and increased the expression levels of Smad3 protein, resulting in an increase in Smad3-mediated transcriptional activity. Further, endogenous BRCA1 associated with Smad3 protein in a TGF-*β*1-dependent manner. The oxidative stress reagent H_2_O_2_ increased the TGF-*β*1-induced BRCA1 and Smad3 endogenous interaction. Of note, in BRCA1-mutated HCC1937 cells, the TGF-*β*1-induced Smad3 and Smad4 interaction was decreased in a dose-dependent manner upon H_2_O_2_ treatment, while reconstitution of wild type BRCA1 in HCC1937 cells restored the TGF-*β*1-induced Smad3 and Smad4 interaction. In addition, the TGF-*β*1-phosphorylated Smad3 protein was sensitive to H_2_O_2_ treatment in HCC1937 cells, resulting in nuclear export of phospho-Smad3 protein, which may contribute to the decrease in its association with Smad4 protein.

We observed that wild type BRCA1 associates with and increases the expression levels of Smad3 protein, resulting in Smad3-mediated reporter activity and an increase in Smad3 and Smad4 association *in vitro*. Compared to Smad4 protein, which significantly augmented Smad3-mediated reporter activity, BRCA1 moderately increased Smad3-mediated transactivation, implying that wild type BRCA1 regulates Smad3-mediated transcription by an alternate mechanism involving Smad4. BRCA1, via the N-terminal region (298–436 aa), associated with Smad3 protein. In functional assays, mutants of BRCA1 could not activate the TGF-*β*1-responsive reporter or the expression levels of Smad3 protein, suggesting that inactivation in BRCA1 impairs TGF-*β* signaling. Mutations in BRCA1 are often seen in BRCA1-associated breast and ovarian cancers, indicating that genetic mutations in BRCA1 are linked to loss of function in Smad3-mediated TGF-*β* signaling. One of the biochemical functions of BRCA1 is E3 ubiquitin ligase activity, which was shown to ubiquitinate and stabilize its binding partners [23]. Whether the observation that wild type BRCA1 increases Smad3 protein expression is due to wild type BRCA1-mediated ubiquitination of Smad3 protein remains to be investigated.

To support our data regarding the BRCA1 and Smad3 interaction, we sought an inducer for the BRCA1 and Smad3 association. We found that H_2_O_2_ increases the TGF-*β*1-induced BRCA1 and Smad3 endogenous association, suggesting that the association of BRCA1 and Smad3 occurs during signaling cross-talk between TGF-*β* and oxidative stress. BRCA1 activates or inhibits many nuclear transcription factors through protein-protein interactions. In addition, BRCA1 was shown to act as a scaffold protein or utilize its E3 ubiquitin ligase activity to regulate nuclear proteins. Thus, BRCA1 regulated the interactions of Phospho-Smad3 and its cellular localization.

H_2_O_2_-activated ERK signaling was reported to increase the association between Smads and the Sp1 transcription factor, which is required for p21WAF1/Cip1 expression by TGF-*β*1 [24]. Here, we found that H_2_O_2_ increases the TGF-*β*1-induced Smad3 and Smad4 interaction in HaCaT cells, suggesting that H_2_O_2_ may induce p21WAF1/Cip1 expression through regulating Smad3 and Smad4 interaction. However, upon H_2_O_2_ treatment, the TGF-*β*1-induced Smad3 and Smad4 interaction was markedly decreased in a dose-dependent manner in BRCA1-mutated HCC1937 cells. Of note, wild type BRCA1-reconstituted HCC1937 cells were insensitive to increasing amounts of H_2_O_2_, resulting in restoration of the TGF-*β*1-induced Smad3 and Smad4 interaction. This suggests that wild type BRCA1 plays an important role in regulating the Smad3 and Smad4 association during oxidative stress responses. Furthermore, H_2_O_2_ treatment induced the nuclear export of TGF-*β*1-phosphorylated Smad3 protein in HCC1937 cells, but not in HCCBRCA1, MCF-7, COS-7 or HaCaT cells. Knockdown of endogenous BRCA1 in HCCBRCA1 cells resulted in a reduction in the basal levels of phospho-Smad3 protein in the nucleus and induced the nuclear export of phospho-Smad3 protein upon H_2_O_2_ treatment. These data suggest that loss of BRCA1 causes a loss of nuclear phospho-Smad3 protein upon H_2_O_2_ treatment, which may contribute to a decrease in the TGF-*β*1-induced Smad3 and Smad4 interaction.

The mechanism by which H_2_O_2_ induces the nuclear export of phospho-Smad3 remains elusive. ROS are reported to activate MAP kinase [25], PKC [26], and Akt [27], which were shown to regulate Smad3 protein activity [28]–[31]. Whether these kinases are involved in the nuclear export of Smad3 protein is under investigation. Several studies have suggested that the durability of Smad proteins in the nucleus is also regulated by nuclear import/export machinery [32]. The nuclear export components CRM1 (chromosome region maintenance 1) and exportin 4 were demonstrated to mediate the nuclear export of Smad proteins [33]–[35]. In addition, Smad-interacting proteins also affect the retention of Smads in the cytoplasm and nucleus by interfering with the nuclear import or export machinery. FoxH1 (forkhead box H1) and ATF2 (activating transcription factor 2) block nuclear export of Smad2 or Smad3 by inhibiting the interaction of Smads with nuclear export components. FoxH1 competes with the nucleoporin Nup153 for binding to the MH2 region of Smad2, inhibiting Smad2 export from the nucleus [36]. Of note, wild type BRCA1 binds to the MH2 domain in Smad3, the same site for exportin 4 and Nup153 binding, offering the possibility that BRCA1 competes with the nuclear export components for binding to the MH2 domain in Smad3. The H_2_O_2_-induced BRCA1 and Smad3 interaction may contribute to the inhibition of oxidative stress-induced nuclear export of phospho-Smad3 protein, avoiding loss of the Smad3 and Smad4 interaction.

BRCA1 was shown to induce antioxidant expression, while loss of BRCA1 resulted in overproduction of ROS in cultured cells [7]. Recently, Cao *et al.* demonstrated that *Brca1*-deficient mice produce excessive ROS and are sensitive to oxidative stress [8], indicating that BRCA1 plays important roles in ROS production and oxidative stress responses. Loss of BRCA1 is known to impair DNA-damage repair. Our studies demonstrate that wild type BRCA1 associates with and modulates Smad3 protein activity during oxidative stress responses, acting as a regulator of oxidative stress-sensitive proteins. Thus, inactivation of BRCA1 also affects cellular signaling pathways during oxidative stress responses. Several studies have revealed that BRCA1 modulates transcription factors by coupling with environmental stresses. In response to hypoxia, wild type BRCA1 increases the nuclear accumulation of HIF-1α, resulting in vascular endothelial growth factor (VEGF) expression and secretion [37]. In addition, wild type BRCA1 induces the transcription of antioxidants through the Nrf2 transcription factor during oxidative stress responses [7].

Loss of growth inhibition by TGF-*β* occurs early in breast cell transformation. Our observations suggest that inactivation of BRCA1 may result in a reduction in Smad3-mediated growth inhibitory activity during oxidative stress responses. Understanding of whether the pro-oncogenic activity of TGF-*β* signaling is increased during oxidative stress responses in BRCA1-deficient cells is of great interest. Given that BRCA1-deficiency causes excess ROS generation *in vivo*, our data suggest that ROS downregulate ROS-sensitive signaling molecules. With the BRCA1 genetic defect as a model system, underlying cross-talk between developmental signals and environmental factors may help elucidate the breast cancer risk factors in breast cancer initiation and progression.

## Materials and Methods

### Cell culture

HEK293, HEK293T, MCF-7, HaCaT, and COS-7 cells were purchased from American Type Culture Collection (ATCC, Manassas, VA, USA). The BRCA1-mutated HCC1937 breast cancer cell line and wild type BRCA1-reconstituted HCC1937 cells (HCCBRCA1) were kindly provided by Dr. Chen [38]. All cell lines were maintained in RPMI-1640 medium supplemented with 10% FBS (Atlanta Biologicals, Norcross, GA, USA), 2.9 mg/ml Glutamine and 100 U/ml Penicillin/Streptamycin, and incubated in a 5% CO_2_ incubator at 37°C.

### Plasmids and materials

Mammalian expression plasmids for wild type and mutated forms of BRCA1 and bacterial expression plasmids encoding GST-BRCA1 protein fragments were previously described [20]. Smad-related constructs were previously described [39], [40]. Deletion mutants of Smad3 constructs were generated by inserting PCR products into a pCMV-Flag vector (Sigma, St. Louis, MO, USA). The HA-Smad4 plasmid was generated by inserting the PCR product of Smad4 into a pcDNA3 vector [20]. All constructs generated after PCR amplification were confirmed by sequencing.

Anti-HA monoclonal antibody (HA.11) and anti-Flag monoclonal antibody (M2) were purchased from Covance (Denver, PA, USA) and Sigma, respectively. Anti-Smad4 monoclonal antibody, anti-Lamin B1 polyclonal antibody, and anti-Myc monoclonal antibody (9E10) were purchased from Santa Cruz (Santa Cruz, CA, USA). Anti-BRCA1 polyclonal antibody (9010) and anti-BRCA1 C-terminal monoclonal antibody (Ab-3) were purchased from Cell Signaling Technology (Danvers, MA, USA) and Calbiochem (San Diego, CA, USA), respectively. An anti-BRCA1 polyclonal antibody against a 428-683aa fragment of BRCA1, generated in our laboratory, was used for immunostaining. Anti-actin monoclonal antibody was purchased from Chemicon (Temecula, CA, USA). Anti-GAPDH monoclonal antibody was purchased from Abcam (Cambridge, MA, USA). H_2_O_2_ was purchased from Sigma and the TGF-*β*1 ligand was obtained from Chemicon. An anti-Smad3 [pSpS^423/425^] polyclonal antibody (44–246G) from Biosource International (Camarillo, CA, USA) was used for Western blotting. Anti-phospho-Smad3 (Ser^423/425^) (C25A9) polyclonal antibody obtained from Cell Signaling Technology (Danvers, MA, USA) was used for immunostaining. The anti-Smad3 polyclonal antibody was obtained from Upstate (Charlottesville, VA, USA).

### Transfection, immunoprecipitation, and Western blotting

LipofectAMINE plus reagent (Invitrogen, Carlsbad, CA, USA) were used to transiently transfect mammalian cells. At 24 hours after transfection, cells were treated with 2 ng/ml of TGF-β1 with or without H_2_O_2_, as indicated. COS-7 cells were harvested in lysis buffer (25 mM Tris-HCl, pH 7.6, 300 mM NaCl, 1% Triton X-100, 10% Glycerol) containing a protease inhibitor cocktail (Roche, Basel, Switzerland). For endogenous immunoprecipitation, cells treated with 2 ng/ml of TGF-β1, with or without H_2_O_2_, were lysed in HKMG buffer (10 mM Hepes, pH 7.8, 100 mM KCl, 5 mM MgCl_2_, 10% Glycerol, 1 mM DTT, 0.5% NP40) containing a protease inhibitor cocktail. The lysates were pre-cleared by incubation with protein-G Sepharose beads (Amersham Pharmacia, Buckinghamshire, UK) for one hour followed by immunoprecipitation with the indicated antibodies. After four extensive washes with cell lysis buffer, the immunocomplexes were eluted by boiling in sample buffer (125 mM Tris-HCl, pH 6.8, 4% SDS, and 20% glycerol) for 5 min. The samples were separated by SDS-PAGE and transferred onto a PVDF membrane (Millipore, Billerica, MA, USA). The membranes were blocked with 5% nonfat dried milk in PBS-0.1% Tween 20, followed by incubation with primary antibodies overnight at 4°C. The horseradish peroxidase-conjugated secondary antibodies (Amersham Pharmacia, Buckinghamshire, UK) were incubated for 1 hour at room temperature followed by enhanced chemiluminescence treatment (PerkinElmer, Waltham, MA, USA). For total protein extraction, cells were lysed in sample buffer, followed by boiling for 5–10 min.

### GST pull-down assay

The GST pull-down assay was performed with HEK293T total cell lysates transfected with Flag-Smad3 plasmid and 10 µg of bacterially-purified GST-BRCA1 protein fragments, as described [20].

### Luciferase activity assay

COS-7 cells were transfected with the indicated plasmids by using LipofectAMINE plus reagent. The cells were treated with 2 ng/ml of TGF-*β*1 overnight 24 hours after transfection. pCMV*β*-galactosidase plasmid (Clontech, Palo Alto, CA, USA) was used for monitoring transfection efficiency. The luciferase and *β*-galactosidase activities were assayed by using a Luciferase assay kit (Promega, Madison, WI, USA).

### Knockdown of BRCA1 by Small interfering RNA (siRNA)

siRNA against BRCA1 was chemically synthesized (Dharmacon, Chicago, IL, USA). Transfection of siRNAs was performed by using OligofectAMINE reagent (Invitrogen, Carlsbad, CA, USA). The sequence of siRNA-BRCA1 is as follows: 5′-AAC CUG UCU CCA CAA AGU GUG-3′ [41]. The control siRNA-GL2 was purchased from Dharmacon.

### Confocal assay

4×10^4^ of HCCBRCA1 cells were plated on 8-well glass slides (Lab-TekII Chamber Slide system) (Nalge Nunc International, Rochester, NY, USA). Cells were treated with 2 ng/ml of TGF-*β*1 and 300 µM of H_2_O_2_ for 1 hour as indicated. Cells were fixed with 3% paraformaldehyde/PBS for 10 minutes and permeabilized with 0.5% Triton X-100/PBS for 5 minutes. The permeabilized cells were blocked with 10% goat serum, 0.1% Triton X-100 in PBS for 2 hours, followed by incubation with anti-BRCA1 (Ab-3) monoclonal antibody and anti-phospho-Smad3 polyclonal antibody overnight. Cells were washed three times with 0.1% Triton X-100/PBS for 30 minutes, and then anti-mouse Rhodamine-conjugated IgG and anti-Rabbit FITC-conjugated IgG antibodies (Jackson ImmunoResearch Laboratories, West Grove, PA, USA) were added (diluted at 1∶250). After 1 hour, cells were washed three times with 0.1% Triton X-100/PBS for 30 minutes, followed by mounting. Images were analyzed using a Zeiss LSM 5 image examiner at the Harvard Center for Neurodegeneration and Repair.

#### Statistical analysis

Data are reported as the mean±standard error of the mean (SEM). The Student's *t* test was used to assess the significance of three independent experiments. *P*<0.05 was used as the criterion to determine statistical significance.
